# Fresh-Cooked but Not Cold-Stored Millet Exhibited Remarkable Second Meal Effect Independent of Resistant Starch: A Randomized Crossover Trial

**DOI:** 10.3390/nu16234030

**Published:** 2024-11-25

**Authors:** Xiyihe Peng, Zhihong Fan, Jinjie Wei, Rui Liu, Xinling Lou, Jiahui Hu, Yuqing Xing

**Affiliations:** 1 College of Food Science & Nutritional Engineering, China Agricultural University, Beijing 100083, China; pxyh0831@cau.edu.cn (X.P.); 13312551374@163.com (J.W.); liurui543@163.com (R.L.); xinlinglou@cau.edu.cn (X.L.); hujiahui1023@126.com (J.H.); 2 Key Laboratory of Precision Nutrition and Food Quality, Department of Nutrition and Health, China Agricultural University, Beijing 100083, China; 3 College of Biological Science and Technology, Beijing Forestry University, Beijing 100091, China; xingyuqing16@bjfu.edu.cn

**Keywords:** glycemic response, second meal effect, millet, slowly digestible starch, resistant starch

## Abstract

It is well established that cold storage results in increased resistant starch and a reduced glycemic index in carbohydrate food. However, the effects of cold storage on the glycemic response of the second meal of cereals remain unclear. The aim of this study was to compare the postprandial glycemic responses between the paired glutinous and non-glutinous grains, either fresh-cooked or refrigerated, after both the first and second meals. In this randomized crossover trial, eighteen healthy female participants consumed eight test meals, each containing 50 g of carbohydrate, including fresh-cooked non-glutinous and glutinous rice, non-glutinous and glutinous millet, and their refrigerated counterparts (4 °C for 24 h). Postprandial blood glucose and insulin were measured at 240 min and 120 min after breakfast. After a standard lunch, the participants’ blood glucose concentrations were measured within 180 min. The rapidly digestible starch (RDS), slowly digestible starch (SDS), and resistant starch (RS) contents of the samples were determined by in vitro enzymatic analysis. Cold-stored non-glutinous rice (CR) and cold-stored non-glutinous millet (CM) had a 24.4% and 29.5% lower incremental area under the curve (iAUC_glu_) of glucose within 240 min compared to the control (fresh-cooked rice non-glutinous, FR), respectively (*p* < 0.05). There were no significant differences between either the cold or hot glutinous grains and FR with respect to postprandial glycemic and insulinemic parameters. After a standard lunch, the fresh-cooked non-glutinous millet (FM) achieved a 39.1% lower iAUC_glu0–180_ compared to the FR (*p* < 0.05). FM had the highest percentage of SDS (64.8%, *p* < 0.05) among all grain samples. Refrigeration treatment reduced the glycemic excursion only in non-glutinous grains at the first meal, but the FM instead of CM demonstrated a significant second meal effect.

## 1. Introduction

According to the International Diabetes Federation (IDF), the number of patients with diabetes was expected to reach approximately 783 million by 2045, while individuals with impaired glucose tolerance (IGT) and impaired fasting glucose (IFG) were estimated to grow to 730 million and 441 million, respectively [1]. With appropriate lifestyle intervention, the prediabetic population can slow or reverse the process of progressing to diabetes and reduce their risk of cardiovascular diseases and early mortality [2]. Dietary strategies to minimize glycemic variability are one of the key measures for diabetes prevention and metabolic health maintenance [3].

As the most important source of carbohydrates in daily diets, grain foods have substantial impacts on glycemic management due to the disparity of the types and characteristics of the carbohydrate components [4], as well as the digestibility of starch after preparation [5,6]. Understanding the behaviors and patterns of grain foods in terms of postprandial glycemic responses is crucial for diabetes prevention and public health management.

When gelatinized starch is cooled at low temperatures for an extended period, the process of retrogradation starts as the amylose double helices aggregate to form a B-type crystalline thermostable structure, i.e., the type 3 resistant starch (RS3). At the same time, the slowly digestible starch (SDS) increases, and the rapidly digestible starch (RDS) decreases [7]. These changes in starch fractions resulted in slowed carbohydrate digestion and led to lowered postprandial blood glucose and insulin responses [8,9].

In some studies, the increase in resistant starch due to cold storage has been shown to be associated with a lowered glycemic index (GI) [7,10] and attenuated glycemic and insulinemic response [11,12]. However, the differences between refrigerated glutinous and non-glutinous grains in terms of glycemic behavior are still limited. What is more, research on the “second meal effect” of fresh-cooked and refrigerated foods is rarely reported.

The “second meal effect” refers to the phenomenon that the ingestion of some low-GI or fiber-rich foods in the first meal resulted in delayed carbohydrate digestion and absorption, reduced insulin secretion [13], promoted short-chain fatty acid (SCFA) production [14,15], and significantly improved glycemic variation in the subsequent meal.

The mechanism of the second meal effect was previously explained by the colon fermentation of resistant starch and some fibers and the production of SCFAs [16,17]. There is a possibility that the cold storage treatment of starchy food might enhance the second meal effect and achieve a glycemic improvement over a two-meal period by providing RS3.

Therefore, the aim of this study was to compare the postprandial glycemic responses between the paired glutinous and non-glutinous grains, either fresh-cooked or refrigerated, after both the first and second meals. The starch fractions, including RS, RDS, and SDS of rice and millet samples, were determined using in vitro enzymatic assays to explore the possible association between these starch fractions and glycemic behaviors. In the present study, we use rice and millet as test materials due to their widespread global consumption and their significance for populations at high risk of diabetes. Intake of polished rice was reported to be positively associated with diabetes risk [18], while millet was regarded as a good choice in glycemic management [19]. The paired glutinous and non-glutinous rice and millet were tested for comparison in the trial because, theoretically, the non-glutinous type of cereals will form a substantial amount of RS after cold storage, while the glutinous type will not. We hypothesized that the types of starch, as well as the serving temperature, might play a role in the glycemic responses of the food samples and pose impacts beyond the first meal and extend to the second meal.

## 2. Materials and Methods

### 2.1. Participants and Ethics

Current students at China Agricultural University were recruited as participants through social media advertisements and were included if they met the following criteria: physically fit females with a body mass index (BMI) between 18.5 and 24.9 kg/m^2^, a regular sleep-wake cycle, a habit of eating breakfast, and regular three meals a day at approximately 8:00, 12:00, and 18:00.

The following exclusion criteria were used to screen potential participants: allergy or intolerance to any of the test foods, being reluctant to eat cold foods, not consuming any grains for more than 1 week, a change in body weight of more than 5 kg in the past 6 months, diabetes mellitus, hypertension, metabolic syndrome, gastrointestinal disorders, such as gastroesophageal reflux, heavy alcohol consumption, regular cigarette smoking, use of medications or supplements known to interfere with metabolism, and participation in competitive or endurance sports.

According to the previous studies and the pilot trials conducted in the laboratory, a minimum sample size of 16 was required in order to observe a difference in the iAUC between treatments with an 80% power at a 5% significance level. The number of participants recruited was increased to 24 as an expected attrition rate of 20% was taken into account. The power calculation was conducted using the PASS software (version 21.0.3, NCSS LLC., Kaysville, UT, USA).

All participants were required to attend the laboratory three days prior to the start of the test sessions for anthropometry measurements of physical parameters, including height, weight, body fat, visceral fat index, basal metabolism, blood pressure, waist circumference, and hip circumference, and were required to fill out a Dutch Eating Behavioral Questionnaire (DEBQ) to assess whether or not they were adopting extreme dietary behaviors. The fat mass was measured using the body fat scale (HBF-371, OMRON, Yangzhou, China), and the basal metabolism rate (BMR) and visceral fat indices were obtained in the meantime. Resting blood pressure was measured in duplicate with an electronic blood pressure monitor (HEM-7200, OMRON, Dalian, China), and the waist-hip ratio was measured with tape. In addition, all screened participants participated in an oral glucose tolerance test (OGTT), and those who met the following criteria were finally included in the trial: fasting glucose < 6.1 mmol/L, peak glucose < 10.0 mmol/L, and 2 h glucose < 7.8 mmol/L.

All data were collected in the College of Food Science and Engineering, China Agricultural University. The study protocol was conducted in full compliance with the Helsinki Declaration, granted by the Ethics Committee of China Agricultural University (protocol code CAUHR-20231206 and date of approval was 13 December 2023), and registered on the Chinese Clinical Trial Registry (ChiCTR2400081460). All participants signed a written informed consent form. The intervention will be terminated if participants report serious adverse reactions or health problems.

### 2.2. Study Design

The randomized crossover trial (RCT number: ChiCTR2400081460) consisted of two groups of independent test sessions, i.e., the fresh-cooked and the refrigerated meals. Each group included four grain meals made of (1) non-glutinous rice, (2) glutinous rice, (3) non-glutinous millet, and (4) glutinous millet, each containing 50 g of available carbohydrate. The cold-stored samples were refrigerated at 4 °C for 24 h. There was at least a one-day washout period between two test sessions. Participants were randomly allocated to the eight test meals according to the result provided by a computer software program (http://www.randomizer.org). Participants were masked to randomization order until after baseline measures were completed. All tests avoided the menstrual period of female subjects.

The participants were instructed not to consume alcohol or sugar-filled meals or beverages, to go to bed no later than 00:00, to avoid intense exercise, and to have breakfast, lunch, and dinner at 8:00, 12:00, and 18:00 during the study period. Due to the nature of the test food, blinding was not possible for either the subjects or the food server. However, the statistician remained blinded to the treatment assignments during the initial data collection.

#### 2.2.1. Postprandial Blood Glucose Concentration Monitoring

Subjects wore Abbott continuous glucose monitors (CGMs; Shanghai, China) the day before the test started (Day 0). A 24 h observation period was scheduled to give subjects time to become used to the sensors and stabilize the glucose monitor. The evening before any test day, at 18:00, participants attended the lab for a standardized dinner. After that, they were asked not to consume any food or drink except water.

After an overnight 13 h fast, the participants arrived at the lab at 7:50, and their baseline blood samples were taken. At 8:00, the subjects received a bowl of cold-stored or freshly cooked grain (each containing 50 g of carbohydrates) and 200 mL of warm water for breakfast. All test meals were required to be consumed within 20 min, and the water needed to be consumed within 120 min after the meal. The time to take the first bite of food was defined as 0 min. Postprandial blood glucose concentrations were measured at 0, 15, 30, 45, 60, 90, 120, 150, 180, 210, and 240 min. A standardized lunch consisting of rice, chicken, lettuce, yogurt, and salad dressing was provided to participants at 12:00 (240 min after breakfast). Participants were required to finish their lunch within 20 min, and blood glucose concentrations were measured at 15, 30, 45, 60, 90, 120, 150, and 180 min after lunch.

#### 2.2.2. Blood Collection and Metabolite Measurements

Capillary blood samples were collected from participants’ fingertips right before the start of breakfast (0 min) and at 15, 30, 45, 60, 90, and 120 min after breakfast and transferred into EDTA K2-treated centrifuge tubes (WanDGL Ltd., Jinan, China). The volume of the blood samples was 150 μL. The samples were centrifuged at 1000× *g* for 15 min within 30 min after blood collection, and then 60 µL of plasma from the supernatant was dispensed into 0.5 mL Eppendorf tubes and stored at −80 °C before analysis. Insulin concentrations were determined using an ELISA kit (JunLB Ltd., Beijing, China). 

### 2.3. Test Meals

#### 2.3.1. Grain Treatment

Glutinous and non-glutinous rice and millet samples were purchased from the same online store. Both the glutinous and non-glutinous rice were the short-grained japonica type produced in northeastern China. The nutrient composition of the four grains is shown in Table 1. All grain samples used in the trial belonged to a uniform batch to avoid inter-batch variation. All grains were cooked according to the recommended rice-water ratio listed on the product package. The glutinous grains were soaked in water for 12 h prior to cooking. The rice-water ratio was 1:1.5 for glutinous rice and non-glutinous and glutinous millet, and 1:1.2 for rice. The fresh-cooked grain samples were prepared at normal pressure using a rice cooker (MB-FZ4086, Midea, Foshan, China) on the morning of the test day, while the cold-stored samples were served in a bowl and refrigerated at 4 °C for 24 h. The chilled rice bowls were kept airtight by sealing the bowls with plastic films to prevent moisture loss.

#### 2.3.2. Standardized Meals

To eliminate the leftover effect of previous food intake on fasting blood glucose, the participants were provided with a standardized dinner the night before the test days (Table 2). To investigate the “second meal effect” of different grains, a standardized lunch was provided on the test days (Table 2).

### 2.4. In Vitro Starch Digestibility

The starch digestibility of grains was assessed following the method of Englyst et al. [20], with some modifications. A total of 1.8 g of pancreatin (P1625, Sigma-Aldrich, St. Louis, MO, USA) was dispersed in 30 mL of water and centrifuged at 3000 rpm for 10 min. A 27 mL portion of the supernatant was then transferred to a beaker. A total of 60 µL of amyloglucosidase (A7095, Sigma-Aldrich, St. Louis, MO, USA) was diluted to 5 mL, and 3.0 mL of this diluted amyloglucosidase solution was added to the beaker containing the pancreatin solution to create an enzyme mixture. The mixture was freshly prepared for the digestion analysis.

Cooked grains were blended with an equal amount of water using a Midea High-Performance Blender (Midea Group, Guangdong, China), and a sample equivalent to 1 g of dry grains was placed in a 50 mL polypropylene screw-capped centrifuge tube. To each tube, four glass beads (1 cm in diameter), 20 mL of sodium acetate buffer (0.1 M, pH 5.2), and 2.5 mg of guar gum were added. The mixture was thoroughly combined and then incubated in a shaking water bath at 37 °C and 206 rpm for 20 min. Subsequently, 5 mL of the enzyme mixture (pancreatin and amyloglucosidase) was added, and the incubation continued in the shaking water bath at 37 °C and 206 rpm. Samples of the hydrolysate (0.2 mL) were taken prior to enzyme addition and at 20 and 120 min afterward. Each sample was mixed with 4 mL of anhydrous ethanol to stop the reaction. The glucose released was measured using a glucose oxidase assay kit (A154-1-1, Jiancheng, Nanjing, China). By measuring the glucose content in the enzymatic hydrolysate at 0, 20, and 120 min, the amounts of RDS (digested within 20 min), SDS (digested between 20 and 120 min), and RS (undigested after 120 min) were calculated. The results were expressed as a percentage of the total starch content for each type of starch.

### 2.5. Data Processing and Statistical Analysis

Postprandial glucose data were analyzed based on values of change relative to fasting concentrations. The incremental area under the curve (iAUC) for postprandial glucose and insulin responses above baseline concentrations was calculated using the trapezoidal method. Glycemic variability parameters included the incremental peak values (∆Peak_glu_) for postprandial glucose and the large amplitude of glycemic excursions (LAGE). The index of postprandial insulin resistance (HOMA-PP), defined as iAUC_glu0–120_ × iAUC_ins0–120_/22.5, and the index of insulin sensitivity, defined as 2/(mean glucose × mean insulin + 1) [21], were calculated. 

All the statistical analysis was performed using GraphPad Prism version 8.3.0 for Windows (GraphPad Software, San Diego, CA, USA). The fresh-cooked rice was set as the control group. A two-way repeated measures ANOVA was used to assess the effects of treatment, time, and the interaction of treatment and time. To analyze the differences between the iAUCs and variability parameters, a one-way ANOVA and Duncan’s multiple range test were applied, and Friedman’s test was used for non-normally distributed results. The variables are presented as either the mean ± standard deviation (SD) or the mean value with standard error (SE), with *p* < 0.05 considered statistically significant.

## 3. Results

### 3.1. Participants Characteristics

**Figure 1:**
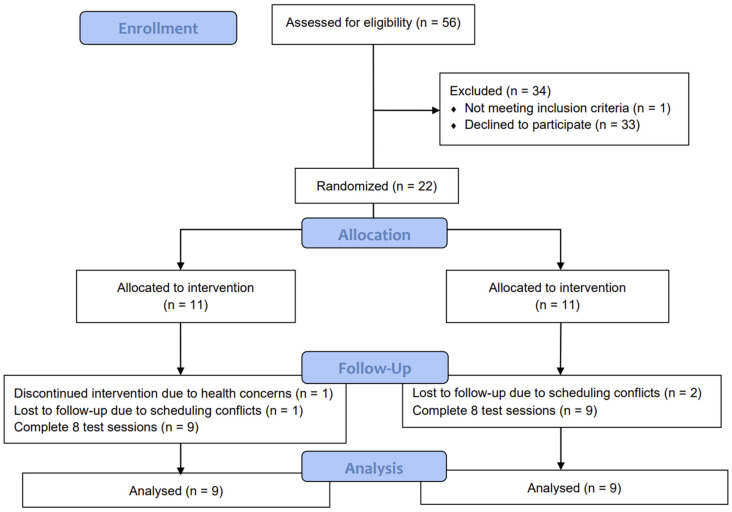
Consolidated standards of reporting trial (CONSORT) flow diagram of the study participants.

A total of 22 female subjects were screened and enrolled in the test. Three withdrew due to scheduling conflicts and one due to health concerns, resulting in a total of eighteen participants completing all procedures; their test data were included in the analyses (Figure 1). There were no significant differences in fasting glucose or insulin among the participants. No subjects reported any adverse reactions or unpleasant sensations throughout the test (Table 3).

### 3.2. Postprandial Glycemic Response for the First Meal and the Second Meal

The participants’ glucose concentrations were continuously monitored for 420 min, covering the 240 min following the test meals and the 180 min after a standardized lunch. There were no significant differences in baseline (i.e., 0 min) blood glucose levels.

**Figure 2:**
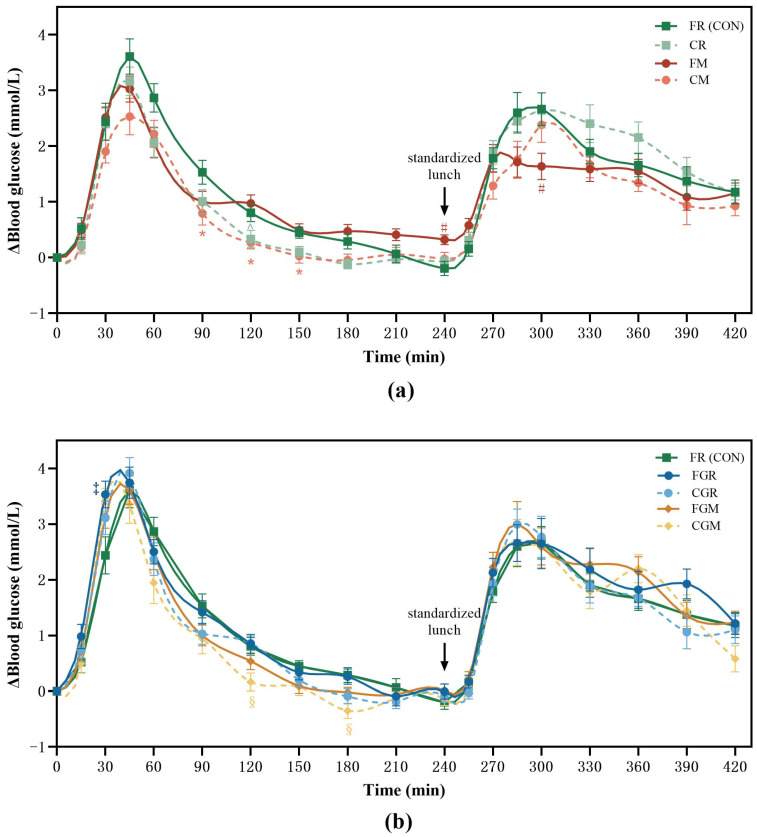
Postprandial glycemic curves from 8:00 to 15:30. FR, fresh-cooked non-glutinous rice; CR, cold-stored non-glutinous rice; FM, fresh-cooked non-glutinous millet; CM, cold-stored non-glutinous millet; FGR, fresh-cooked glutinous rice; CGR, cold-stored glutinous rice; FGM, fresh-cooked glutinous millet; CGM, cold-stored glutinous millet. Data show the mean ± SE (*n* =18). * CM differ from FR counterparts; ^#^ FM treatments differ from FR counterparts; ^§^ CGM differ from FR counterparts; ^‡^ FGR differ from FR counterparts (*p* < 0.05). (**a**) Postprandial glycemic curves of non-glutinous grain; (**b**) Postprandial glycemic curves of glutinous grain.

With respect to the first meal, compared to fresh-cooked rice (FR), the cold-stored non-glutinous millet (CM) induced significantly lower blood glucose responses at 90, 120, and 150 min (0.79, (SE 0.2) mmol/L, *p* = 0.0493; 0.27 (SE 0.1) mmol/L, *p* = 0.0237; 0.03 (SE 0.1) mmol/L, *p* = 0.0326, respectively) (Figure 2a), while the cold-stored glutinous millet (CGM) resulted in lower blood glucose concentrations at 120 and 180 min (0.17, (SE 0.2) mmol/L, *p* = 0.0272; −0.35 (SE 0.1) mmol/L, *p* = 0.0083, respectively). The fresh-cooked glutinous rice (FGR) had significantly higher blood glucose concentrations at 15 min than FR (0.98 (SE 0.2) mmol/L, *p* = 0.0404) (Figure 2b).

Regarding the second meal, the fresh-cooked non-glutinous (FM) had significantly higher blood glucose concentrations prior to the start of lunch (0.32 (SE 0.1) mmol/L, *p* = 0.0067) than FR. Compared with HR, the FM showed significantly lower blood glucose concentrations at 60 min (1.64 (SE 0.2) mmol/L, *p* = 0.0067) and a trend towards significantly lower blood glucose concentrations at 45 min after lunch (1.72 (SE 0.2) mmol/L, *p* = 0.069) (Figure 2a). There were no significant differences among the other test meals at all time points.

**Figure 3:**
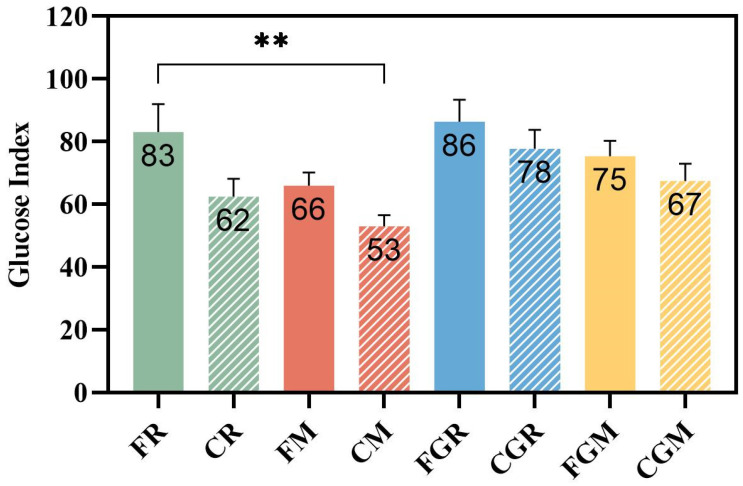
The glucose index of test grains. FR, fresh-cooked non-glutinous rice; CR, cold-stored non-glutinous rice; FM, fresh-cooked non-glutinous millet; CM, cold-stored non-glutinous millet; FGR, fresh-cooked glutinous rice; CGR, cold-stored glutinous rice; FGM, fresh-cooked glutinous millet; CGM, cold-stored glutinous millet. Data show the mean ± SE (*n* = 18). ** *p* < 0.01.

To examine the effect of the test meals on glycemic stability metrics, the GI and iAUC were calculated. As expected, the non-glutinous rice and both fresh-cooked glutinous grains were high-GI food. However, despite the cold storage, glutinous rice remains a high-GI food, whereas rice, glutinous rice, and millet achieve medium to low GI. Compared with the corresponding freshly cooked samples, the GI values of refrigerated rice and millet decreased by 24.8% and 20.6%, respectively. Although the extent of GI reduction in cold-stored non-glutinous samples was greater than that observed in the glutinous grains (10.5% for glutinous rice and 10.7% for glutinous millet), they failed to reach statistical significance. Compared with FR, only CM achieved a significant GI reduction (*p* = 0.0029) (Figure 3). Compared with FR, significant decreases were observed in the iAUC for CR and CM (24.4% (*p* = 0.0131); 29.5% (*p* = 0.0123)) within 240 min after the first meal (Table 4).

After consuming the same standardized lunch, the group that consumed FM at breakfast had the lowest glycemic response after lunch. Compared with FR, the FM had a significantly lower iAUC over 0~120 and 0~180 min after lunch (reduced by 40.1% and 39.1%, respectively, *p* < 0.001). The CM also had a significantly lower iAUC than FR 180 min after lunch (*p* = 0.0015). Taking the first and the second meal together, CM had the lowest iAUC_glu0–420_, which was significantly different from FR (*p* = 0.0123) (Table 4).

With respect to the glycemic excursion after the first meal, the CM elicited the lowest ΔPeak_glu_ and significantly lower than that of FR (*p* = 0.0354). No significant reduction in ΔPeak_glu_, as well as LAGE_0–240_, was observed among cold storage groups compared with their fresh-cooked counterparts. For the second meal, the ΔPeak_glu_ (*p* = 0.0032) and LAGE_0–180_ (*p* = 0.0036) of FM was the lowest and significantly lower than that of FR. Combining the results over two meals, the LAGE_0–420_ of FM and CM was significantly lower than that of FR (*p* = 0.0036, *p* = 0.043) (Table 5).

### 3.3. Postprandial Insulinemic Response for the First Meal

**Figure 4:**
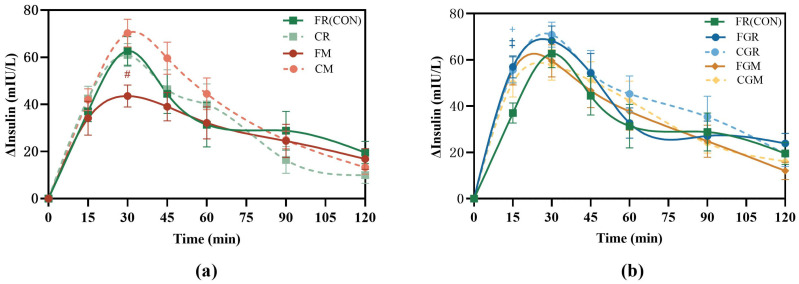
Postprandial insulin curves from 8:00 to 10:00 (**a**,**b**); FR, fresh-cooked non-glutinous rice; CR, cold-stored non-glutinous rice; FM, fresh-cooked non-glutinous millet; CM, cold-stored non-glutinous millet; FGR, fresh-cooked glutinous rice; CGR, cold-stored glutinous rice; FGM, fresh-cooked glutinous millet; CGM, cold-stored glutinous millet. Data show the mean ± SE (*n* = 18). ^#^ FM treatments differ from FR counterparts; ^‡^ FGR treatments differ from FR counterparts; ^+^ CGR treatments differ from FR counterparts (*p* < 0.05).

Compared with FR, the FM elicited significantly lower insulin concentrations (43.30 (SE 5.6) mIU/L, *p* = 0.0366) at 30 min after meal (Figure 4a), while both the CGR and HGR induced significantly higher insulin concentrations (55.64 (SE 5.6) mIU/L, *p* = 0.0451; 56.97 (SE 4.7) mIU/L, *p* = 0.0139) at 15 min after meal (Figure 4b).

**Figure 5:**
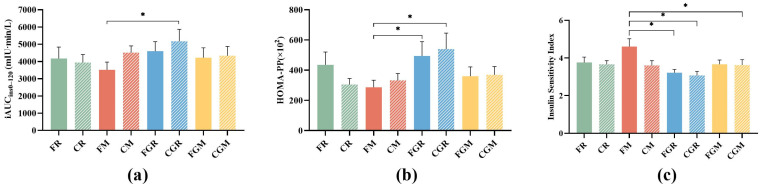
Postprandial capillary insulin iAUC from 8:00 to 10:00 (**a**), HOMA-PP (**b**) and insulin sensitivity index (**c**); FR, fresh-cooked non-glutinous rice; CR, cold-stored non-glutinous rice; FM, fresh-cooked non-glutinous millet; CM, cold-stored non-glutinous millet; FGR, fresh-cooked glutinous rice; CGR, cold-stored glutinous rice; FGM, fresh-cooked glutinous millet; CGM, cold-stored glutinous millet. Data show the mean ± SE (*n* = 18). * *p* < 0.05.

Among the eight test meals, the FM showed the lowest iAUC_ins0–120_ (Figure 5a) and HOMA-PP (Figure 5b), while the CGR had the highest. The FM also ranked first in terms of ISI (Figure 5c) but had no significant difference with FR and CM.

### 3.4. Starch Digestibility

**Figure 6:**
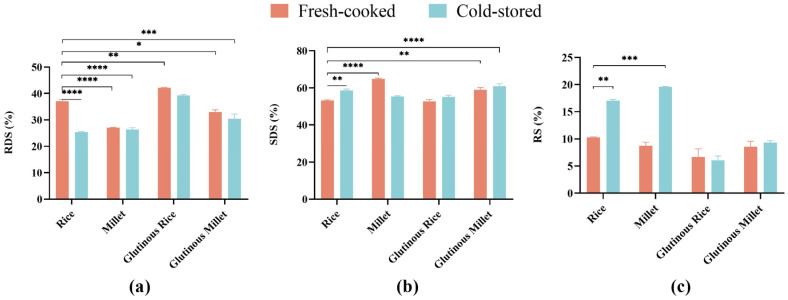
(**a**) Proportion of rapidly digestible starch to total starch in different cereals; (**b**) proportion of slowly digestible starch to total starch in different cereals; (**c**) proportion of resistant digestible starch to total starch in different cereals. * *p* < 0.05, ** *p* < 0.01, *** *p* < 0.001, **** *p* < 0.0001.

The starch digestibility of different test meals is shown in Figure 3. There were significant differences (*p* < 0.05) in the proportions of RDS, SDS, and RS among the eight grain meals. The FGR showed the highest proportion of RDS, significantly higher than all other test groups except CGR. There was no significant difference in the proportion of RDS between either the FM and CM or FGR and CGR, while the RDS proportion of CR was significantly lower than that of FR (Figure 6a).

FM had the highest proportion of SDS among the eight groups, with significant differences compared with FR and the other test groups (*p* < 0.05). There was no significant difference in SDS proportion among rice, glutinous rice, and glutinous millet regardless of the refrigeration treatment (Figure 6b).

CM exhibited the highest proportion of RS among the eight groups, followed by CR. Refrigeration treatment significantly boosted the RS fractions in both non-glutinous rice and millet but not their glutinous counterparts (Figure 6c).

## 4. Discussion

This study compared the effect of refrigeration on paired non-glutinous and glutinous grains (rice and millet) on postprandial blood glycemic and insulinemic responses, as well as the possible second meal effect. In this study, as expected, the refrigeration treatment had less impact on glutinous grains. However, interestingly, the result showed that fresh-cooked millet, instead of the cold-stored millet, had a significant second meal effect the other test meals.

Judging by the GI figure, the glycemic responses of the samples were reduced after cold storage. However, the cold storage had less impact on the immediate postprandial blood glucose and insulin responses in the two glutinous grains compared with their non-glutinous counterparts. This difference can be explained by the amylose content of the samples. Previous studies have shown that starchy grains high in amylose tend to elicit an attenuated blood glucose response after being stored at low temperatures due to the increase in RS3 [22].

In an in vitro digestion study by Li et al. [7], cold storage at 4 °C effectively reduced the estimated glycemic index (eGI) of white rice compared to freshly cooked white rice. A recent study found that compared to those stored at room temperature, wheat products stored at 4 °C for 24 h exhibited a lowered glycemic index and glycemic load in healthy individuals (43, 32.3 vs. 41.1, 28.6) [12]. Similarly, a study by Strozyk et al. on 32 patients with type 1 diabetes found that long-grain white rice refrigerated for 24 h caused significantly lower blood glucose peaks, incremental glucose peaks, and AUCs [23]. Patterson et al. compared freshly cooked potatoes with potatoes stored at 4 °C for 5 days and found that postprandial glucose levels dropped by 4.8% at both 15 and 30 min after consuming the refrigerated potatoes [24].

On the other hand, the increase in RS content after cold storage may not associated with a noticeable reduction in postprandial blood glucose. Sissons et al. [25] reported that the in vitro digestibility of pasta did not change significantly after refrigeration treatment, even when the resistant starch content of pasta increased by about 37%. Likewise, a clinical trial by Hodges et al. [26] found no difference in the glycemic response AUC between refrigerated and freshly cooked pasta.

In the present study, glutinous grains were included as test meals to confirm the role of RS in mitigating the glycemic response of refrigerated carbohydrates. After cold storage, the RS contents of non-glutinous rice and millet increased significantly, leading to notably decreased postprandial glycemic responses, as shown by the iAUC_glu0–240_ values for CR and CM. However, the RS content of glutinous grains remained almost unchanged after cold storage, resulting in an insignificant reduction in their postprandial glycemic responses.

In addition to examining the postprandial blood glucose and insulin responses, the second meal effect of fresh-cooked and refrigerated grains was also investigated in the present study. The second meal effect was first introduced by Jenkins et al., who found that ingesting low GI food at the first meal could improve the glucose tolerance of the subsequent meal [13]. The carbohydrate sources that have been reported to show potential second meal effects, including barley products [27], quinoa, buckwheat [28], lentils, and chickpeas [29], are grains rich in dietary fiber. A commonly accepted explanation of the second meal effect is that the dietary fiber and RS provide substrates for colon fermentation and enhance the production of SCFAs, which help to improve insulin sensitivity [30].

However, Brighenti et al. found that, when ingested with fermentable dietary fiber, even a high-GI meal rich in RDS could produce a beneficial second meal effect similar to that induced by a low-GI meal [17]. This suggests that the second meal effect is not strictly dependent on the GI of the first meal. The key point might be the presence of fermentable carbohydrates because they can improve glucose metabolism by providing the non-esterified fatty acids (NEFAs) to compete with glucose for energy substrate [17]. Additionally, fermentable carbohydrates may improve glucose metabolism and insulin sensitivity by modifying the level of serum free fatty acids (FFAs) [31], delaying gastric emptying and promoting intestinal motility [32]. Therefore, supplementing fermentable carbohydrates is considered a dietary strategy to elicit the second meal effect. For instance, consuming a drink containing added barley β-glucan at breakfast has been shown to be able to reduce post-lunch blood glucose levels [33].

In refrigerated non-glutinous grains, the amylose-initiated retrogradation results in the formation of RS3, which can be fully fermented in the colon and augment the production of SCFAs. Sanders et al. found that even though there was no significant difference in insulin sensitivity after consuming potatoes stored at 4 °C for 15 h compared to a meal with equal calories, fasting blood glucose levels the next morning were lower following a single intervention [34]. We surmised that there might be the possibility that the glycemic stabilizing effect could carry over to the second meal.

However, the present study found that, although the cold storage did significantly increase the amount of RS in CR and CM, it was the fresh-cooked millet, instead of the cold-stored millet or rice, that elicited the most stable glycemic response after the second meal. The capacity of second meal effect seemed to have no association with the content of RS and dietary fiber, both of which was rather low in fresh-cooked millet.

We found similar results in previous studies. Oba-Yamamoto et al. [16] developed a low-starch, high-fiber pasta with added resistant dextrin, which significantly lowered blood glucose levels during the first meal but did not exhibit a second meal effect. In another study, compared with bread made from regular flour, the bread made with 85% and 70% high-amylose starch resulted in significantly lower postprandial glycemic responses at the first meal. However, there was no difference in blood glucose response after a standard lunch [35]. MacNeil et al. [36] reported that while high RS-content food effectively decreased blood glucose levels after the first meal, it did not necessarily result in a stronger second meal effect. Kinnear et al. [37] found that cold potatoes slowed digestion but failed to trigger a significant second meal effect

According to the timeline of digestion, it takes up to 48 h from food ingestion to the completion of colon fermentation of undigested food components [30]. In our study, the fresh-cooked millet showed a particularly pronounced glycemic mitigating effect at 4 to 5 h after consumption. The time gap between breakfast and lunch (4 h) may be too short for producing enough SCFAs to directly and substantially impact the post-lunch glucose metabolism. We suppose that the glycemic benefits of RS might limited to the first meal and might not carry over to the second meal.

Another possible mechanism of the second meal effect involves the effects of organic acids on free fatty acids, gastric emptying rate, and incretins [38,39]. However, in the present study, no organic acid was used in the test meals.

Based on the starch fraction analysis, there is the possibility that the SDS might play a crucial role in the second meal effect, which has not been widely reported before. Unlike indigestible RS, SDS is broken down and absorbed in the small intestine, but at a slower rate [5]. Studies have shown that foods high in SDS can extend the glucose release profile [40], providing a more sustained and balanced energy supply [41]. Unlike RDS, SDS induces a delayed but stable prolonged glucagon-like peptide-1 (GLP-1) and glucose dependent insulinotropic peptide (GIP) response, starting from 3–5 h after consumption, which just fit the timeline in the present trial.

It is noticed that, although the iAUC_glu0–240_ for the first meal showed no significant difference among the eight test meals, at the time point of 240 min (used as the baseline for the second meal), only the FM had a significantly higher glucose level than FR. It may not be a coincidence that the highest glucose level at 240 min and the lowest LAGE_240–420_ were observed in the FM. We surmised that this result could be explained by a slow and gradual glucose release of FM due to the appropriate proportion of RDS and SDS, which optimized the release of glucose regulation hormones.

SDS not only controls blood glucose by slowing the absorption of exogenous glucose but also reduces endogenous glucose production. Péronnet et al. [42] found that compared to a low-SDS diet, a high-SDS diet helped to stabilize postprandial blood glucose levels by reducing the production of endogenous glucose. As a sustained-release source of glucose, SDS ingestion creates a more effective negative feedback loop and reduces hepatic glucose production (EGP). At the same time, since SDS slows the rate of appearance of exogenous glucose (RaE), the demand for insulin secretion is decreased, leading to mitigated glycemic and insulinemic response and improved insulin sensitivity [43].

In the current study, although the FM did not show significantly lower blood glucose levels during the first meal, it did have a 30.5% lower insulin concentration at 30 min post-meal compared to the FR, and it achieved the highest insulin sensitivity among the eight test meals. This hypothesis may explain the strong second meal effect exhibited after the FM meal and deserve further testing.

In this study, glutinous grains were also included as test meals. In China, glutinous rice is typically categorized as a food to be consumed cautiously by individuals with impaired glucose metabolism. Compared with refined foods, whole grains are generally believed to be beneficial to glucose and insulin homeostasis in healthy individuals. However, it is not confirmed whether the glutinous whole grains would be low-glycemic food [44]. Nakayama et al. [45] found that an 8-week intervention replacing white rice with glutinous brown rice significantly improved glycated hemoglobin levels in patients with type 2 diabetes.

In this study, the glycemic and insulin responses elicited by glutinous millet and glutinous rice were not significantly different from those of rice in either the first or second meal. In spite of the fact that FGM had a similar SDS content to FM, the FGM failed to exhibit a potent second meal effect as FM did. We suppose that the high RDS content might masked the SDS’s effect by causing an early glucose spike. According to Eelderink et al. [46], blood glucose levels do not always reflect the actual digestion rate of starch in the body, as the rate of glucose release during digestion affects how efficiently tissues can absorb glucose. While the starch digestion rates vary across foods, the glucose absorption by tissues may also modified by food components as well. This food-blood glucose kinetics needs further in-depth research.

The strength of this study lies in its dual perspective on the impact of cold storage on the postprandial glycemic response and the second meal effect of grains. First, it examined the differences in starch digestibility alterations induced by cold storage between paired non-glutinous and glutinous grains. Second, it investigated the sustained impact of grain test meals on glycemic variability in the second meal—an aspect not addressed in earlier studies. Possible confounding factors that could reduce the reliability of the study were minimized or well-controlled. The first meal consisted solely of grains to eliminate the potential impact of other nutrients on the outcomes. For the second meal, the same standardized lunch was provided to all participants to avoid variations in nutrient composition that could affect the results.

The first limitation is that the results are derived from healthy young female participants rather than a more diverse group. The reasons for recruiting healthy young females as participants include the following: (1) The standard lunch is a crucial component in evaluating the second meal effect. To avoid potential variations in the second meal effect caused by different portion sizes of the standard lunch, it was necessary to ensure that participants had similar daily food intake and metabolic levels. The healthy young females included in this study were similar in ethnicity, age, height, weight, basal metabolic rate, and appetite. (2) Less male participants expressed interest, while some of them had the habit of smoking, alcohol drinking, and strenuous physical activity. Single-gender volunteers were chosen to ensure sufficient eligible participants while maintaining the consistency of metabolic and intake levels described in (1). It is confirmed that the gender of subjects does not significantly affect glycemic and insulin responses, as age, ethnicity, and pathological conditions do [47,48,49,50]. Although the number of participants in this study was sufficient for data analysis, including more participants with diverse backgrounds would enhance the generalizability of the findings

The second limitation is that, considering the acceptability of the subjects, blood collection after 120 min was not performed to measure changes in insulin concentration over a 4–5 h of time. Hormones and metabolic markers of SCFAs, GIP, and GLP-1 during both the first and second meals were not analyzed. In addition, this study was designed as an acute intervention. The long-term effect of grain consumption needs to be confirmed in well-designed intervention studies.

## 5. Conclusions

In this study, well-controlled randomized trials were conducted to compare the postprandial blood glucose and insulin responses, as well as the second meal effects of eight test grain meals. The results showed that non-glutinous grains with a higher content of amylose showed an increase in RS contents after cold storage, leading to a lower glycemic response but not insulin response after the first meal. Fresh-cooked millet demonstrated a remarkable second meal effect, which might be associated with its high proportion of SDS instead of RS. Cold storage treatment did not produce significant differences in glycemic and insulin responses in glutinous grains.

Notably, millet’s potential as a healthy food was reaffirmed, as the cold-stored millet led to a lowered blood glucose response at the first meal, while the fresh-cooked millet contributed to improved glycemic stability after the second meal.

The results of the present study suggest that the second meal effect may not depend solely on the GI of the first meal and the RS fraction. Further exploration of the second meal effect of carbohydrate food and the possible mechanism has implications for guiding dietary choices in glycemic management.

## Figures and Tables

**Table 1:** The nutrient composition of the test grains.

Grain	Carbohydrate (g/100 g)	Protein (g/100 g)	Fat (g/100 g)	Fiber (g/100 g)	Amylose (g/100 g)
Non-glutinous rice (R)	76.7	6.6	0.6	0.7	15.7
Glutinous Rice (GR)	77.6	9.5	2.5	1.0	3.0
Non-glutinous millet (M)	73.0	9.0	3.1	1.8	10.8
Glutinous Millet (GM)	70.6	13.6	2.7	1.9	0.6

**Table 2:** The composition, macronutrient, and energy contents of the standardized meals.

Standardized Meals	Carbohydrate (g)	Protein (g)	Fat (g)	Energy (kcal)	Detail Content
Lunch	83.3	19.6	15.0	547	uncooked rice 80 g, instant chicken breast 35 g, roasted sesame dressing 25 mL, lettuce 50 g, yogurt 180 mL
Dinner	82.6	19.6	15.1	545	uncooked rice 80 g, instant chicken breast 30 g, mixed vegetable salad dressing 25 mL, lettuce 50 g, yogurt 180 mL

**Table 3:** Baseline characteristics of study participants (*n* = 18).

Characteristics	Mean ± SD
Age (years)	22.7 ± 1.1
Body composition	
BMI (kg/m^2^)	21.4 ± 2.0
Fat mass (%)	26.3 ± 3.0
Waist: hip ratio	0.7 ± 0.0
Visceral fat index	2.8 ± 1.1
Basal metabolic rate (kcal/day)	1264.3 ± 87.5
Physical examination	
Fasting blood glucose level (mmol/L)	4.8 ± 0.4
Fasting insulin level (mIU/L)	14.6 ± 6.7
Systolic blood pressure (mmHg)	109.6 ± 6.9
Diastolic blood pressure (mmHg)	65.7 ± 6.4
Eating behavior	
Rice intake per meal (g)	61.1 ± 20.8
External eating score	3.3 ± 0.4
Emotional eating score	2.5 ± 0.8
Restrained eating score	2.7 ± 0.7

**Table 4:** Incremental area under the postprandial glucose curve for the first and second meals (mean ± SE, *n* = 18).

	First Meal		Second Meal		First + Second Meal
Test Meals	iAUC_glu0–120_ (mmol·min/L)	iAUC_glu0–240_ (mmol·min/L)	iAUC_glu240–360_ (mmol·min/L)	iAUC_glu240–420_ (mmol·min/L)	iAUC_glu0–420_ (mmol·min/L)
FR	219.9 ± 18.1	281.4 ± 22.6	232.8 ± 21.8	328.8 ± 26.0	583.2 ± 37.0
CR	179.7 ± 16.4	212.6 ± 16.1 **	239.0 ± 20.0	338.5 ± 25.2	546.4 ± 40.7
FGR	230.0 ± 18.9	300.5 ± 18.5	223.2 ± 25.7	326.2 ± 34.7	637.3 ± 46.1
CGR	216.8 ± 16.1	270.6 ± 20.5	253.2 ± 21.6	347.8 ± 25.6	571.6 ± 32.5
FM	175.9 ± 15.2	247.8 ± 16.6	139.3 ± 15.4 ***	200.1 ± 17.9 ***	495.6 ± 36.0 *
CM	167.2 ± 16.2	198.3 ± 16.4 **	177.9 ± 18.7 *	251.0 ± 21.4 ***	449.4 ± 37.2 **
FGM	188.0 ± 17.1	254.9 17.1	244.8 ± 20.8	338.1 ± 27.4	592.1 ± 40.8
CGM	191.6 ± 16.7	240.9 ± 21.0	238.6 ± 26.6	335.3 ± 33.4	553.8 ± 49.8

FR, fresh-cooked non-glutinous rice; CR, cold-stored non-glutinous rice; FM, fresh-cooked non-glutinous millet; CM, cold-stored non-glutinous millet; FGR, fresh-cooked glutinous rice; CGR, cold-stored glutinous rice; FGM, fresh-cooked glutinous millet; CGM, cold-stored glutinous millet. * *p* < 0.05, ** *p* < 0.01, *** *p* < 0.001. The time point 0 was set as the start of breakfast and the 420 min after breakfast was 180 min after lunch.

**Table 5:** Glycemic fluctuation parameters after the first and second meal (mean ± SE, *n* = 18).

	First Meal		Second Meal		First + Second Meal
Test Meals	ΔPeak_glu_ (mmol/L)	LAGE_0–240_ (mmol/L)	ΔPeak_glu_ (mmol/L)	LAGE_240–420_ (mmol/L)	LAGE_0–420_ (mmol/L)
FR	3.8 ± 0.3	4.2 ± 0.4	3.5 ± 0.3	3.6 ± 0.3	4.5 ± 0.3
CR	3.2 ± 0.2	3.7 ± 0.3	3.3 ± 0.2	3.4 ± 0.3	4.1 ± 0.3
FGR	4.1 ± 0.2	4.3 ± 0.3	3.7 ± 0.3	3.8 ± 0.3	4.6 ± 0.3
CGR	4.1 ± 0.3	4.6 ± 0.3	3.8 ± 0.3	4.0 ± 0.3	5.0 ± 0.2
FM	3.4 ± 0.2	3.4 ± 0.2	2.3 ± 0.2 ***	2.5 ± 0.2 **	3.6 ± 0.2 **
CM	3.0 ± 0.2 *	3.5 ± 0.2	2.9 ± 0.2	3.0 ± 0.2	3.8 ± 0.2 ***
FGM	3.9 ± 0.2	4.3 ± 0.3	3.8 ± 0.3	3.8 ± 0.3	4.8 ± 0.3
CGM	3.9 ± 0.3	4.5 ± 0.3	3.6 ± 0.3	3.7 ± 0.3	4.7 ± 0.3

FR, fresh-cooked non-glutinous rice; CR, cold-stored non-glutinous rice; FM, fresh-cooked non-glutinous millet; CM, cold-stored non-glutinous millet; FGR, fresh-cooked glutinous rice; CGR, cold-stored glutinous rice; FGM, fresh-cooked glutinous millet; CGM, cold-stored glutinous millet. * *p* < 0.05, ** *p* < 0.01, *** *p* < 0.001.

## Data Availability

The data presented in this study are available upon request from the corresponding author. The data are not publicly available due to privacy.
